# Case Report: Type II tyrosinemia caused by mutations at the c.843_844 inv p.(Trp282Gly) variant locus

**DOI:** 10.3389/fgene.2026.1753440

**Published:** 2026-03-12

**Authors:** Fei Tong, Meirong Peng, Lingzhang Meng, Jiajia Shen, Lin Huang, Weifang Huang, Weitong Huang, Jian Song

**Affiliations:** 1 Department of Clinical Laboratory, Nanning Maternity and Child Health Hospital, Nanning, Guangxi, China; 2 Children’s Health Department, Nanning Maternity and Child Health Hospital, Nanning, Guangxi, China; 3 Research Laboratory, Nanning Maternity and Child Health Hospital, Nanning, Guangxi, China; 4 Institute of Cardiovascular Sciences, Guangxi Academy of Medical Sciences, & The People’s Hospital of Guangxi Zhuang Autonomous Region, Nanning, China

**Keywords:** c.1125 + 1G>T, c.843_844inv, children, TAT gene, tyrosinemia type II

## Abstract

**Objective:**

To identify the genetic etiology in a neonate with persistent hypertyrosinemia and characterize the functional impact of a novel *TAT* gene variant, c.843_844inv.

**Methods:**

A neonate suspected of having tyrosinemia type II following newborn screening by tandem mass spectrometry was recruited, along with family members. Whole-exome sequencing (WES) was performed to identify causative variants. To validate the pathogenicity of the identified novel locus, wild-type and mutant *TAT* expression vectors were constructed. These vectors were transfected into 293T cells to assess mRNA and protein expression levels *in vitro*. Structural modeling was also employed to predict the impact of the variant on protein stability.

**Results:**

The proband exhibited persistently elevated blood tyrosine levels (>600 μmol/L) on repeated screenings. Genetic analysis revealed compound heterozygous variants in the *TAT* gene: a known pathogenic splice-site variant, c.1125 + 1G>T (maternal), and a novel variant, c.843_844inv (p. (Trp282Gly)) (paternal). The proband’s healthy sister carried only the c.843_844inv variant. *In vitro* functional assays demonstrated that while *TAT* mRNA levels were unaffected, the p. (Trp282Gly) mutation significantly reduced *TAT* protein expression to approximately 20.7% of wild-type levels. Structural modeling suggested that the p. (Trp282Gly) substitution disrupts critical hydrogen bonds in the enzyme’s core region.

**Conclusion:**

A novel pathogenic variant, c.843_844inv (p. (Trp282Gly)), was identified in the *TAT* gene, which, in combination with c.1125 + 1G>T, causes tyrosinemia type II. Functional studies confirmed that this novel variant leads to a significant reduction in *TAT* protein levels. These findings expand the mutational spectrum of *TAT* and provide a basis for clinical diagnosis and genetic counseling.

## Introduction

Tyrosinemia type II (Richner-Hanhart syndrome; OMIM #276600) is a rare autosomal recessive disorder caused by a deficiency in hepatic tyrosine aminotransferase (*TAT*), an enzyme encoded by the *TAT* gene located on chromosome 16q22 ^1^. The clinical presentation typically includes elevated plasma tyrosine levels, painful palmoplantar hyperkeratosis, pseudodendritic keratitis, and variable degrees of intellectual disability (Huhn et al., 1998; Thibault et al., 2022). While dietary restriction of phenylalanine and tyrosine can effectively alleviate oculocutaneous symptoms, it imposes a significant burden on patients (Hannah-Shmouni et al., 2019).

Diagnosing tyrosinemia type II prenatally presents a challenge. Unlike some metabolic disorders, tyrosine and its metabolites do not significantly accumulate in amniotic fluid, and the *TAT* enzyme is not expressed in chorionic villi or amniocytes (Maydan et al., 2006). Consequently, molecular genetic analysis remains the gold standard for definitive diagnosis and prenatal testing.

This study investigated a neonate identified with persistent hypertyrosinemia during newborn screening. Through whole-exome sequencing and family segregation analysis, a novel compound heterozygous genotype involving a previously unreported variant, c.843_844inv (p. (Trp282Gly)), was identified. The functional impact of this novel variant was further characterized through *in vitro* expression assays and structural modeling to provide evidence for its pathogenicity.

## Materials and methods

### Study design and oversight

The proband, a male infant, was identified via newborn screening via tandem mass spectrometry (MS) 3 days after birth because of elevated tyrosine levels (601.21 μmol/L; reference range 40–270 μmol/L). Repeat testing at 1 month and 20 days confirmed persistent hypertyrosinemia (640.16 μmol/L). Comprehensive biochemical evaluations were performed, including liver function tests (TBIL, DBIL, ALT, AST, TP, ALB, GGT, ALP, CHE, TBA, and GLDH), renal function tests (CREA, UREA, UA, and CYS-C), lipid profiles, cardiac enzymes, and glucose levels. Urine organic acid analysis was conducted to screen for specific metabolites. We suspected that the child had hereditary tyrosinemia. After written informed consent was obtained from the parents, peripheral blood samples were collected from the proband, parents, and elder sister to investigate the underlying cause of hypertyrosinemia, which would facilitate treatment planning. Whole-genome sequencing revealed that the patient carried compound heterozygous mutations: c.1125 + 1G>T and c.843_844inv. On the basis of persistent hypertyrosinemia in the blood and specific metabolites identified via urinary organic acid analysis, the clinician made a definitive diagnosis of tyrosinemia type II. We implemented dietary restriction of tyrosine intake to maintain the patient’s tyrosine levels within a controlled range, thereby minimizing the adverse effects of elevated tyrosine on growth and development. Follow-up evaluations were conducted at 3 days, 7 weeks, nearly 6 months, 8 months, and 1 year. The child’s growth and development were comparable to those of peers of the same age. According to the WHO Child Growth Standards (2006), the patient’s height and weight reached medium levels, whereas the body mass index (BMI) was at lower-medium levels.

### Sequencing experiments

First, 1 µg of the DNA genome was extracted from 200 µL of whole blood samples interrupted by ultrasound, and DNA was extracted uniformly from all samples via the Qiagen DNA Blood Midi/Mini Kit (Qiagen GmbH, Hilden, Germany). The extraction process was carried out in strict accordance with the manufacturer’s instructions. The 50 ng of DNA was interrupted into a fragment of approximately 200 bp, followed by end repair and 3′end A addition. Then, the DNA fragments were ligated with barcoded sequencing adaptors, and fragments of approximately 320 bp were collected with XP beads. The pre-library was subjected to a liquid hybridization capture operation with reference to the standard procedure for Nano-WES microarrays. The hybridization products were eluted and collected, and then, PCR amplification and purification were performed to obtain the exon library. The library was quantified via qPCR. High-throughput sequencing was performed via the Illumina NovaSeq 6,000 platform (Illumina, San Diego, United States), and raw data were obtained after CASAVA v1.82 processing. The quality of the data was more than 85% of the bases meeting the Q30 or above (≧Q30) standard, more than 95% of the bases meeting the Q20 or above (≧Q20) standard, and the duplication rate was no more than 30%.

### Data analysis

The raw genomic sequencing data were filtered for quality control, the sequenced sequences were aligned to the human reference genome (hg19/GRCh37) via the Burrows-Wheeler Aligner tool, and the repetitive PCR sequences were removed via Picard v1.57 (http://picard.sourceforge.net/). Variant detection analysis was performed via the Verita Trekker® Variant Detection System developed by Berry Genetics and GATK (https://software.broadinstitute.org/gatk/). Variant annotation was performed via ANNOVAR (Wang et al., 2010) and the Enliven® Variant Annotation Interpretation System developed by Berry Genetics. The annotation databases include i) population databases such as gnomAD (http://gnomad.broadinstitute.org/), the 1000 Genomes Project (http://browser.1000genomes.org), the Berry big data population database, and dbSNP (http://www.ncbi.nlm.nih.gov/snp); ii) prediction algorithms such as SIFT (http://sift.jcvi.org), FATHMM (http://fathmm.biocompute.org.uk), Mutation Assessor (http.//mutationassessor.org), CADD (http://cadd.gs.washington.edu), SPIDEX (Xiong et al., 2015), etc.,; and iii) disease and phenotypic databases, such as OMIM (http://www.omim.org), clinvar (http://www.ncbi.nlm.nih.gov/clinvar), HGMD (http://www.hgmd.org), and HPO (https://hpo.jax.org/app/).

SpliceAI and RNA Splicer software were used to predict the pathogenicity of the splice-site mutation, and the known pathogenicity was verified by querying the HGMD and ClinVar databases.

### Gene function

#### c.843_844inv gene wt and mut expression vector construction strategy

##### pCMV-3XFlag-Neo (EGFP)-TAT-wt vector construction

*The* pCMV-3XFlag-Neo(EGFP)-*TAT*-HindIII-F primer 5′gac​aag​ctt​ATG​GAC​CCA​TAC​ATG​ATT​CA3′ and pCMV-3XFlag-Neo(EGFP)-*TAT*-KpnI-R primer 5′gac​tgg​tac​cCT​ATT​TAT​CAC​ACT​CCT​CCT3′, synthesized by Gene Create (Wuhan, China), were used as templates for the whole-gene synthesis of the *TAT* CDS, the HindIII-wt-KpnI fragments were obtained via PCR amplification, the wt fragments of HindIII and KpnI (NEB, United States) were double-cleaved to obtain the HindIII-wt-KpnI fragments, and the pCMV-3XFlag-Neo(EGFP) vector was digested and ligated with the T4 DNA Ligase Kit (Thermo Fisher, United States) to obtain the pCMV-3XFlag-Neo(EGFP)-*TAT*-wt vector. The pCMV-3XFlag-Neo (EGFP) vector was ligated after digestion to obtain the pCMV-3XFlag-Neo (EGFP)-*TAT*-wt vector, which was sequenced and verified.

##### pCMV-3XFlag-Neo (EGFP)-TAT-mut vector construction

The mut-1 fragment was obtained via PCR via whole-gene synthesis with the *TAT* CDS as a template, the pCMV-3XFlag-Neo (EGFP)-*TAT*-HindIII-F primer and the *TAT* mut-R primer, 5’agc​cag​gaa​cca​gcc​ctc​gct​tgg​cca​gcc​ct3′, synthesized by Gene Create (Wuhan, China), and the mut-fragment was amplified via PCR via the *TAT*-mut-F primer 5’agg​gct​ggc​caa​gcg​agg​gct​ggg​ttc​ctg​gct3′ and the pCMV-3XFlag-Neo (EGFP)-*TAT*-KpnI-R primer. 2 fragments. A second round of PCR amplification was performed using mut-1 and mut-2 as templates and pCMV-3XFlag-Neo (EGFP)-*TAT*-HindIII-F and pCMV-3XFlag-Neo (EGFP)-*TAT*-KpnI-R as primers to obtain HindIIImut-KpnI fragments. After sequencing to verify that the mutation has been introduced and that other sequences are correct, Hind III and KpnI (NEB, United States) double-cut the mut fragment and pCMV-3XFlag-Neo (EGFP) vector, ligated them enzymatically with a T4 DNA Ligase Kit (Thermo Fisher, United States) to obtain the pCMV-3XFlag-Neo (EGFP) *TAT*-mut vector, and then sequenced with primers for verification.

The constructed *TAT*-wt and *TAT*-mut vectors were subjected to Sanger sequencing (ABI 3730xl sequencer, Thermo Fisher), and the sequencing results were aligned with the wild-type *TAT* CDS via DNAMAN 9.0 software to confirm the successful introduction of the c.843_844inv mutation and the corresponding amino acid change (Trp282→Gly).

### Cell transfection

293T cells, purchased from the Cell Bank of the Chinese Academy of Sciences (Shanghai), were cultured in DMEM containing 10% fetal bovine serum and 1% penicillin/streptomycin (Gibco) at 37 °C with 5% CO2 and 95% O2. The cells were inoculated into six-well plates 1 day prior to transfection and transfected the next day when the cells reached 70%–90% confluence. Then, 5 µL of Lipofectamine™ 3,000 reagent (Invitrogen) was added to each well with 125 µL of Opti-MEM and mixed well. DNA premix was prepared by diluting 2.5 µg of DNA per well with 125 µL of Opti-MEM. P3000™ reagent was added and mixed well, diluted DNA (1:1 ratio) was added to each tube of diluted Lipofectamine™ 3,000 Reagent, and the mixture was incubated for 10–15 min at room temperature. The DNA‒lipid complex was incubated with the cells, mixed gently, and incubated in an incubator.

### qPCR assay

To detect the relative mRNA expression level of the *TAT* gene in 293T cells, adherent 293T cells were digested with 0.25% trypsin-EDTA (Gibco, United States) and collected 48 h after transfection with the wild-type and mutant eukaryotic recombinant expression vectors. Total RNA was extracted with TRIzol reagent (Invitrogen), and cDNA was reverse transcribed with the PrimeScript RT Reagent Kit (TaKaRa). The primers *TAT*-qPCR-F 5’agc​cat​tgt​gga​caa​cat​ga3′ and *TAT*-qPCR-R 5’agcttctagggcctca3′ were used to detect the expression levels of the wild-type and mutant transcripts in the pCMV-3XFlag-Neo (EGFP) vectors via a qPCR system (SYBR Green Master Mix, Roche).

### Western blot analysis

The cells were lysed in RIPA buffer containing protease inhibitors. The protein concentration was determined via a BCA protein assay kit (Yeasen, China). Lysates (30 μg of total protein per well) were separated via SDS‒PAGE (10% separating gel and 5% stacking gel) and transferred onto 0.45 μm PVDF membranes (Millipore, United States). The membranes were blocked with 5% skim milk and incubated with primary antibodies against GAPDH, Flag, or GFP (1:1,000, Cell Signaling Technology) to detect the expression of *TAT* proteins. Finally, after incubation with secondary antibodies (1:5,000, Beyotime), the blots were analyzed via the chemiluminescence method via the ECL Western blotting Substrate Kit (Thermo Fisher, United States), imaged via a Gel Doc XR + Gel Imaging System (Bio-Rad, United States) and detected via Kodak Digital Science 1D software (Eastman Kodak Co., New Haven, CT, United States). The protein bands were visualized, and the band intensity was quantified from three independent experiments.

### Protein modeling

To evaluate the detrimental effect of the candidate p. (Trp282Gly) variant on the *TAT* protein, we performed a structural bioinformatics analysis. The canonical amino acid sequence of human *TAT* was retrieved from the NCBI protein database and used as the target for homology modeling with the SWISS-MODEL server. The template structure (PDB: 3PDX) was selected to ensure coverage of over 30% of the target sequence and, crucially, to fully encompass the residue at position 282 and its surrounding structural region. The generated three-dimensional model was then visualized and manipulated via SPDBV (version 4.1). Within this software, the wild-type tryptophan at position 282 was mutated to glycine via the residue mutation function, followed by local energy minimization to relieve potential steric clashes. Finally, the hydrogen bond networks in the wild-type and Trp282Gly mutant models were calculated and compared via the built-in analysis tools in SPDBV to identify any loss or alteration of key stabilizing interactions.

## Results

### Clinical characteristics and outcomes of the study participants

The proband presented elevated tyrosine levels (601.21 μmol/L) at 3 days of age. Despite dietary management, the levels remained high at subsequent checks (640.16 μmol/L at 1 month; 603.40 μmol/L at 5 months). Importantly, liver function test results (ALT, AST, TBIL, etc.), renal function, lipid, and glucose levels were consistently within normal ranges (Tables 1, 2). Urinary organic acid analysis revealed no significant abnormalities, and the level of succinylacetone was not elevated, excluding type I tyrosinemia. Clinically, the child had a mild phenotype. At 5 months, development was normal, with no cranial MRI or funduscopic abnormalities. By 1 year, the child had walked independently (3-5 steps) and showed no corneal or skin lesions.

**TABLE 1:** Liver function results at 4 months, 5 months, and 1 year of age.

Inspection item	4 months	5 months	1 year	References range
TBIL	7.70	11.20	4.85	0–23.00 μmol/L
DBIL	2.30	5.30	1.82	0–8.00 μmol/L
ALT	44	41.0	14.00	9–50 U/L
AST	44	38.0	27.40	15–40 U/L
TP	54.4	58.2	63.60	65–85 g/L
ALB	42.1	43	45.60	40–55 g/L
GGT	17	14	11.30	10–60 U/L
ALP	229	275	239.9	0–449 U/L
CHE	​	8,448	8,891	5,000–12000 U/L
TBA	5.7	7.03	1.50	0.5–10 μmol/L

**TABLE 2:** Results for renal function, lipids, and myocardial enzymes at 5 months.

Inspection item	5 months	References range
GLDH	4.1	0–7 U/L
CREA	18	57–97 μmol/L
UREA	1.2	3.1–8.0 mmol/L
UA	258	208–428 μmol/L
CYS-C	1.19	0.51–1.09 mg/L
CHOL	3.66	0–5.18 mmol/L
TG	0.64	0–1.7 mmol/L
LDL-C	2.14	0–3.37 mmol/L
HDL-C	1.53	1.16–1.42 mmol/L
CK	129	50–310 U/L
LDH	217	120–250 U/L
LAC	1.31	0.5–2.20 mmol/L
GLU	5.37	3.89–6.11 mmol/L

#### Gene analysis of the study participants

**FIGURE 1:**
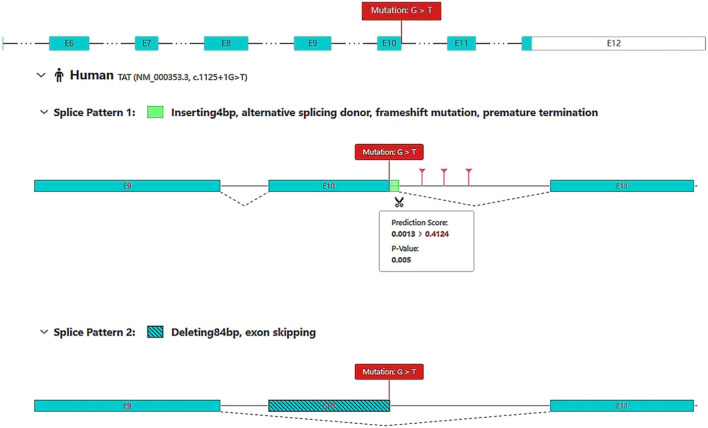
Splice site mutation at the c.1125 + 1G>T locus of the *TAT* gene. The c.1125 + 1G>T locus is predicted to be 0.99 by Splice AI to be deleterious, as predicted by RDDC’s RNA Splicer, which may produce two splicing modes: **(A)** A 4 bp insertion downstream of exon 10, which changes the splicing donor and results in a code-shifting mutation that terminates early; **(B)** exon 10 undergoes a jump.

**FIGURE 2:**
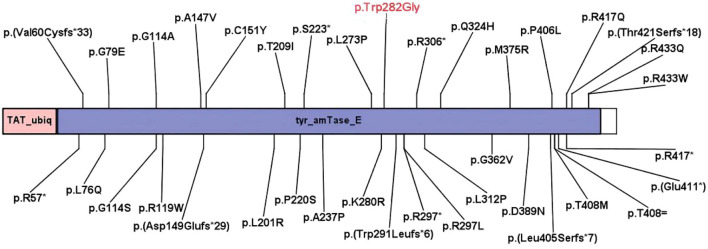
The functional domains predicted by NCBI. Two functional domains, tyr_amTase_E and *TAT*_ubiq, where tyr_amTase_E is the tyrosine aminotransferase functional region, and the tyrosine aminotransferase is the first enzyme in the pathway of tyrosine degradation through homogeneous substances and catalyzes the conversion of L-tyrosine to p-hydroxyphenyl pyruvic acid. Mutations in the tyrosine aminotransferase functional region result in loss of function. *TAT*_ubiq is the ubiquitination site of transaminase. This fragment contains a possible ubiquitination site that ensures rapid degradation of the tyrosine transaminase. *TAT*_ubiq is the ubiquitination site of the tyrosine transaminase.

Sequencing of the child’s blood samples revealed two heterozygous mutations in the *TAT* gene. The first was c.1125 + 1G>T, a splice region mutation known to be potentially pathogenic (Figures 1, 2). Figure 1 shows the predicted abnormal splicing modes of the c.1125 + 1G>T variant (4 bp insertion or exon 10 skipping). Figure 2 shows the functional domains of the *TAT* gene and the distribution of known pathogenic mutations, clarifying the location of the novel p. (Trp282Gly) variant in the tyr_amTase_E functional domain. At 8 months and 9 days, the retested tyrosine concentration was 671.09 μmol/L, and at 1 year and 12 days, it was 638.13 μmol/L. Despite these biochemical abnormalities, the child’s development remained consistent with that of her age-matched peers.

#### Genetics study

**FIGURE 3:**
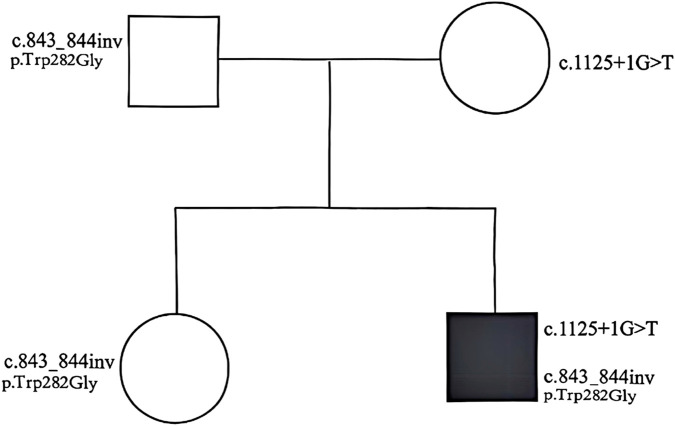
Inheritance map of mutations in the *TAT* gene in the child’s family line. Both the father’s and the mother’s disease-causing genes were affected. The proband carried compound heterozygous mutations (c.1125 + 1G>T heterozygous/c.843_844inv heterozygous), the father and elder sister were heterozygous carriers of c.843_844inv, and the mother was a heterozygous carrier of c.1125 + 1G>T. The proband’s older sister was found to be a carrier of the c.843_844inv heterozygous mutation.

**FIGURE 4:**
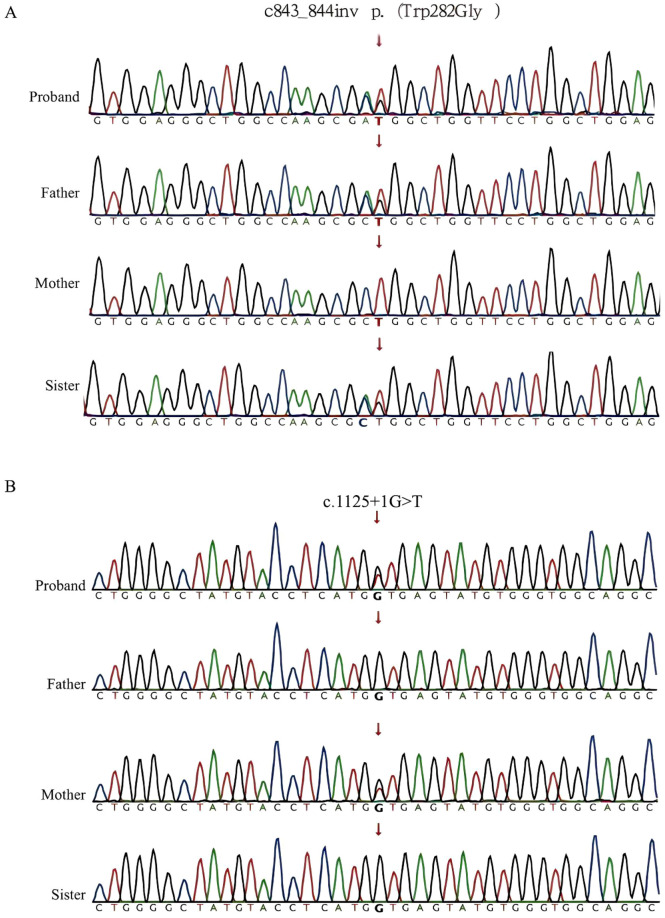
Mutation map. **(A)** Mutation map of the c.843_844inv p. (Trp282Gly) locus of the *TAT* gene in the family line affected by the child; the father of the affected child and the sister of the affected child are indicated by the arrows as heterozygotes carrying the mutation in the c.843_844inv locus of the gene. **(B)** Mapping of the c.1125 + 1G>T locus mutation in the *TAT* gene in the family line of the affected child. The heterozygotes carrying the c.1125 + 1G>T locus mutation in the affected child and the affected mother are indicated by the arrows.

To clarify the cause of the disease, whole-genome sequencing was performed for the entire family. The results confirmed that the proband carried compound heterozygous mutations: c.1125 + 1G>T and c.843_844inv. Segregation analysis revealed that the father carried the c.843_844inv heterozygous mutation, whereas the mother carried the c.1125 + 1G>T heterozygous mutation. The proband’s older sister was a carrier of the c.843_844inv heterozygous mutation (Figure 3). The c.1125 + 1G>T mutation has been demonstrated to cause tyrosinemia, but the mother (a carrier) was phenotypically normal. Database screening (HPO, OMIM, GHR) initially suggested that c.843_844inv was a variant of undetermined significance (Figure 4).

#### Gene function analysis

**FIGURE 5:**
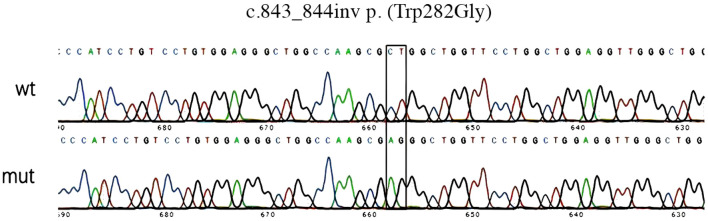
Sequencing results of *TAT*-wt/mut vector construction. If you have already responded to any other reviewers, please submit your response to this reviewer and/or revised manuscript within seven calendar days.

**FIGURE 6:**
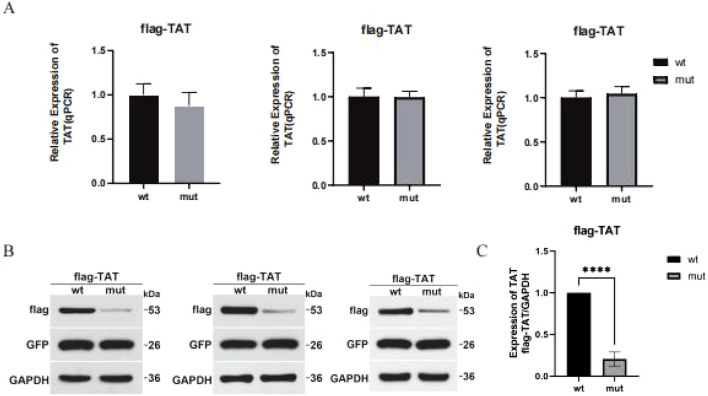
Effect of mutation on *TAT* expression. **(A)** Relative expression of *TAT* mutant and wild-type mRNAs (QPCR). **(B)** Relative expression of the *TAT* mutant and the wild-type protein (WB). **(C)** Quantitative analysis of *TAT* protein expression (n = 3, p < 0.01).

To clarify the function of the c.843_844inv mutation site, gene expression validation was performed. c.843_844inv (p. (Trp282Gly)) caused a change in amino acid 282 from tryptophan to glycine (Figure 5). After the *in vitro* expression assay, the mRNA levels of the mutants in the pCMV-3XFlag-Neo (EGFP) vector did not change significantly compared with those in the wild type (Figure 6A). However, the protein levels decreased significantly. Quantitative analysis of three independent experiments revealed that mutant protein expression was significantly reduced to 20.7% ± 8.4% of the wild-type level (p < 0.01) (Figures 6B,C).

#### Structural alteration of the TAT protein

**FIGURE 7:**
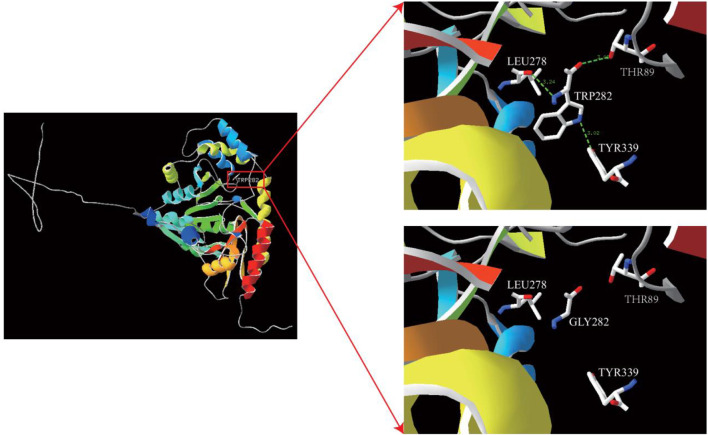
Predicted 3D structure of TRP282 transformed into the GLY282 protein.

The impact of this missense variant was evaluated by mapping the mutation position onto the crystal structure of *TAT* (PDB ID: 3PDX). Three-dimensional structural modeling revealed that the affected residue is located in the core region of *TAT*. In the wild-type protein, residue TRP282 forms three hydrogen bonds with LEU278, TYR339, and THR89 at distances of 3.02 Å, 3.02 Å, and 3.24 Å, respectively. When tryptophan was replaced with glycine, these hydrogen bonds were lost (Figure 7). Protein modeling revealed conformational space changes in the *TAT* variant, which potentially caused the observed instability and functional impairment.

## Discussion

In the present study, significantly elevated tyrosine was detected in one neonate via neonatal screening, leading to a diagnosis of type II tyrosinemia. Further study revealed a novel mutation, c.843_844inv (p. (Trp282Gly)), in this child and his family, which contributes to the disease. This discovery provides a basis for future clinical research and prenatal diagnosis.

The patient was found to have compound heterozygous mutations in the *TAT* gene: c.1125 + 1G>T and c.843_844inv (p. (Trp282Gly)). A literature review confirmed that c.1125 + 1G>T is associated with pathogenic tyrosinemia (Beyzaei et al., 2022). According to ACMG/AMP guidelines (Richards et al., 2015), the novel c.843_844inv variant was re-evaluated. It is absent from population databases (PM2) and was detected in trans with a pathogenic variant (PM3), which is consistent with previous reports where compound heterozygosity was established as a definitive cause of the disease (Legarda et al., 2011). Furthermore, functional investigations conducted in this study demonstrated that the mutation results in the loss of hydrogen bonds between TRP282 and LEU278, TYR339, and THR89. Consequently, the expression of the mutant protein was significantly decreased to approximately 20.7% of the wild-type level, providing supporting evidence of pathogenicity (PS3_supporting). Therefore, c.843_844inv is classified as likely pathogenic. These findings demonstrate that compound heterozygosity for c.1125 + 1G>T and c.843_844inv, which are associated with the *TAT* gene, causes type II tyrosinemia.

Type II tyrosinemia is caused by defects in the *TAT* gene (16q22), which encodes tyrosine transaminase (Beyzaei et al., 2022). This enzyme catalyzes the conversion of L-tyrosine to p-hydroxyphenylpyruvate. The inability to deaminate tyrosine leads to hypertyrosinemia. While ocular and cutaneous symptoms typically manifest, phenotypic variability exists. Barr et al. (1991) reported that controlling tyrosine concentrations between 400 and 800 μmol/L resulted in less affected growth. The present patient presented with tyrosine levels of approximately 650 μmol/L under dietary restriction but presented a mild phenotype without typical corneal or skin abnormalities, and development was consistent with age (Hannah-Shmouni et al., 2019; Chinsky et al., 2017).

The parents and elder sister of the proband were all heterozygous carriers of a single *TAT* mutation and had no hypertyrosinemia or clinical symptoms of tyrosinemia type II, which is highly consistent with the known autosomal recessive inheritance pattern of tyrosinemia type II (OMIM #276600) and further verifies the genetic characteristics of this disease (Legarda et al., 2011). The proband had compound heterozygous mutations (two alleles of the *TAT* gene carried pathogenic mutations), leading to a significant reduction in *TAT* protein expression (only 20.7% of the wild-type level) and complete loss of enzyme function, which confirms that the pathogenic key of tyrosinemia type II is biallelic pathogenic mutations (homozygous or compound heterozygous) in the *TAT* gene; a single allelic mutation results in only a carrier state without clinical phenotypes. The molecular mechanism described herein, where mutations at the intron‒exon junction disrupt the canonical splice donor site, leading to aberrant splicing and the production of truncated, nonfunctional proteins, has been validated in several important studies and represents a classic pathogenic mode of splicing mutations (Oftedal et al., 2023; Weisschuh et al., 2021).

The limitations of this study include the lack of direct enzymatic activity assays; however, protein stability data and structural modeling strongly support pathogenicity. Currently, no pharmacological treatment exists for tyrosinemia type II, and dietary restriction remains the standard. This study identified a novel mutation site, advancing the understanding of the molecular mechanisms and potential therapeutic targets for type II tyrosinemia.

## Data Availability

The datasets presented in this article are not readily available because of ethical and privacy restrictions. Requests to access the datasets should be directed to the corresponding authors.
